# Psychological Impact of False-Positive and Inconclusive Newborn Screening Results on Parents in eastern Morocco

**DOI:** 10.1192/j.eurpsy.2026.11494

**Published:** 2026-07-31

**Authors:** F. Wahoud, S. Essadki, R. Lamsyah, M. A. Lafraxo, R. Boughoufa, R. Amrani

**Affiliations:** 1 Higher Institute of Nursing Professions and HealthTechniques; 2 Laboratory of Maternal, Infant, and Mental Health(LSMIM), Faculty of Medicine and Pharmacy, Oujda; 3 Higher Institute of Nursing Professions and HealthTechniques, Fez; 4 Laboratory of Biology and Health, Faculty of Science, Ibn Tofail University, Kenitra, Morocco

## Abstract

**Introduction:**

Newborn screening (NBS) is a cornerstone of early detection for severe congenital disorders. However, the increasing scope of NBS programs has led to a higher frequency of false-positive (FP) and inconclusive (IC) results, which may generate significant psychological distress among parents. In Morocco, particularly in eastern Morocco, where NBS implementation is still developing, understanding these psychosocial consequences is essential for improving parental support and program acceptance.

**Objectives:**

To assess the psychological and behavioral impact of false-positive and inconclusive NBS results on parents in eastern Morocco.

**Methods:**

A cross-sectional descriptive study was conducted between March 2024 and June 2025 among 120 parents (40 FP, 30 IC, and 50 controls) recruited from public maternity hospitals across eastern Morocco. Standardized questionnaires wereused to measure anxiety (State-Trait Anxiety Inventory, STAI), parental stress (Parenting Stress Scale, PSS), and perceived child vulnerability. Semi-structured interviews complemented the quantitative data to capture parental emotional experiences and perceptions of communication with clinicians.

**Results:**

Meananxiety scores were significantly higher among FP (M = 54.2 ± 8.6) and IC parents (M = 50.8 ± 7.9) compared with controls (M = 38.5 ± 6.4; p < 0.001).

Approximately 68% of FP parents said that they felt an intense fear related to perceived disease severity, and 42% sought repeated medical consultations before receiving confirmatory results.

Perceived child vulnerability remained elevated three months after the confirmation in 37% of FP parents. Qualitative findings revealed limited pre-screening information and anxiety-inducing communication during result announcement.

**Conclusions:**

These findings highlight that false-positive and inconclusive newborn screening results exert a significant psychological impact on parents in eastern Morocco. Strengthening communication strategies, enhancing healthcare provider training, and establishing a structured psychological support system are essential to reduce this distress and reinforce parental trust in the national newborn screening program.

**Disclosure of Interest:**

None Declared

